# Puerperal Fever and Erysipelas

**Published:** 1857-11

**Authors:** R. V. Duncan

**Affiliations:** York County, Pa.


ART. VI.—*Puerperal Fever of an Erysipelatous Character treated with Iron and large Doses of Opium.* By R. V. DUNCAN, York County, Pa.

THE following details of a case of childbed fever of a malignant character, will, I hope, prove interesting to the profession, and contribute to the discovery or adoption of some more successful mode of treating this most fatal form of disease. The fatality which has followed the course of treatment generally pursued by medical men, should lead the inquiring practitioner to look for some more successful mode of combating this fatal malady,—some remedy or remedies that would not exhaust the already debilitated lying-in patient; for the idea of debility is associated with every case of labor, as daily observation will prove to every practitioner. And is it good policy on the part of medical men, when their childbed patients fall victims to a rapidly fatal and destructive malady, to drain from the system a large quantity of blood, thus depriving it of that vital fluid which it so much needs to sustain it through the ravages of the disease? The details of the case I am now about to cite will show conclusively that this form of disease can be successfully treated by other means than those of a rigid antiphlogistic character.

On the morning of the 9th of April last, I was called to attend Mrs. K., in confinement with her third child. Her labor was easy, and soon over; the child being born before I arrived at the house. I soon proceeded to remove the secundines very carefully, which passed away in due time. After giving the infant the necessary attention, I took my leave of the patient, who expressed herself as feeling very comfortable indeed.

I visited her the next day, and found her doing very well. On the second and third days I called to see her, and found her very comfortable, and improving rapidly in strength. I discharged myself on the third day, remarking to her at the same time, that she was more comfortable than the most of patients are at that period after confinement.

On the morning of the fifth day, I was summoned in haste to see Mrs. K. Upon arriving at the house, I found her laboring under all the symptoms of puerperal fever. Acute pain; great tenderness, and enormous tympanitic distension of the peritoneal region; pulse one hundred and ten; confusion of ideas; great thirst, and a large erysipelatous blush on the right cheek. After deliberating for some time, I concluded to pursue a different course of treatment from what is generally recommended in those cases, and treat the symptoms as they presented themselves. My patient was suffering much pain; I gave her large doses of morphia at short intervals to overcome that symptom. As soon as the pain was somewhat mitigated, I gave her a dose of calomel, and followed it up with castor-oil and turpentine, which operated in the course of a few hours. I observed, however, that the purgative did not act until I brought the patient fully under the use of the opiate. This fact is worthy of consideration, for it is reasonable to suppose that the high grade of inflammatory excitement existing in the uterine and peritoneal regions, accompanied with violent pain, would necessarily interfere with the peristaltic motion in the bowels, and hence the great difficulty in those cases in being able to evacuate the intestinal canal. As soon as a motion was procured from the bowels, I put the patient on the tincture of the muriate of iron, twenty drops every two hours, and continued the opiate as long as she complained of pain and tenderness. Large, hot poultices were kept constantly applied to the abdominal and pelvic regions. I also made use of a solution of the sulphate of iron to the erysipelatous blush on the cheek, of the strength of an ounce of the salt to a pint of water. My reasons for using the iron were, that I have found, from past experience in several cases, that the preparations of this metal possess a remarkable influence over disease of an erysipelatous character. Under this treatment she commenced to improve on the second day, and continued to improve daily till the fifth day, when the puerperal symptoms subsided, and she was again comfortable, to my great satisfaction, and to the joy and comfort of the family, who had given up all hope of her ever being restored.

On the third day of her illness, my attention was called by the nurse to the infant. She informed me that it had moaned all night, and appeared to be in much pain. Upon removing the dressing from the funis, I found that the same form of disease had set in there. There was the same erysipelatous blush that appeared on the cheek of the mother. I at once gave the little sufferer up, concluding from the reports which I had read of cases of this character that it was hopeless. However, I determined to do something to relieve its pangs. I commenced the use of a weak solution of the sulphate of iron, as an external application to the inflamed and swollen funis, and with a very happy effect. In twenty-four hours after, I observed a favorable change; and, in the course of two days, it was so much improved that I considered it out of danger. By the fifth day it was entirely well. Both mother and child are still living, and in blooming health. Whatever virtue there may be in the course of treatment pursued in this case, I will leave the facts and result to future demonstration.

